# Introducing the keyconcept approach to the analysis of language: the case of regulation in COVID-19 diaries

**DOI:** 10.3389/frai.2023.1176283

**Published:** 2023-09-21

**Authors:** Justyna A. Robinson, Rhys J. Sandow, Roberta Piazza

**Affiliations:** ^1^School of Media, Arts and Humanities, University of Sussex, Brighton, United Kingdom; ^2^Department of Language and Linguistic Science, University of York, York, United Kingdom

**Keywords:** keyconcept, semantic variation, corpus, discourse analysis, COVID-19, regulation

## Abstract

Using the Mass Observation corpus of 12^th^ of May Diaries, we investigate concepts that are characteristic of the first coronavirus lockdown in the UK. More specifically, we extract and analyse concepts which are distinctive of the discourses produced in May 2020 in relation to concepts used in the 10 previous years, 2010–2019. In the current paper we focus on the concept of regulation, which we identify through a novel approach to querying semantic content in large datasets. Typically, linguists look at keywords to understand differences between two datasets. We demonstrate that taking the perspective of a *keyconcept* rather than the *keyword* in linguistic analysis is a beneficial way of identifying trends in broader patterns of thoughts and behaviours which reflect lived-experiences that are particularly prominent of a given dataset, which, in this current paper, is the COVID-19 era dataset. In order to contextualise the keyconcept analysis, we investigate the discourses surrounding the concept of regulation. We find that diarists communicate collective experience of limited individual agency, surrounded by feelings of fear and gratitude. Diarists' reporting on events is often fragmented, focused on new information, and firmly placed in a temporal frame.

## 1. Introduction

In 2020, with COVID-19 spreading across the world population, individuals were forced to adapt to a new reality quickly and dramatically. Changes in social practises included new behaviours such as social-distancing, face-mask wearing, home-working, and many others. These behavioural and often concomitant attitudinal changes happened in real time (see Barber and Kim, 2021; Naughton et al., 2021; Schnell et al., 2021; Woodrow and Moore, 2021). For example, Kleitman et al. (2021) found that perceptions of the severity of the threat of COVID-19, vulnerability to infection, and the efficacy of protective behaviours were highly predictive of the uptake of behaviours relating to the prevention, avoidance, and management of illness. The language used during the COVID-19 era can provide insight into these profound and far-reaching changes that resulted from the pandemic directly or indirectly. Linguistic research has explored public health messaging from the government and related agencies (e.g., Kalocsányiová et al., 2021; Strange, 2022) and the media (Jaworska, 2021; Müller et al., 2021; Semino, 2021; Yu et al., 2021; Kania, 2022; Bafort et al., 2023; Giorgis et al., 2023). A number of studies analyse COVID-19 signage communication, such as Tragel and Pikksaar (2022) and Bagna and Bellinzona (2023). The current paper contributes to the developing body of work that is concerned with the language used during the pandemic by the general public rather than institutions (see also Cowie et al., 2022; Wilding et al., 2023).

In the current paper we explore lockdown language through lexis as it is the layer of language most sensitive to social changes (Minkova and Stockwell, 2009). In previous work we have demonstrated the benefits of investigating changes in society through the lens of lexical variation (e.g., Robinson, 2010, 2012; Sandow and Robinson, 2018; Sandow, 2022, 2023). We did this through exploring different words expressing the same meaning (formal onomasiological perspective), and different meanings expressed by the same word (semasiological perspective). In the current work we complement these approaches by building on conceptual onomasiology (Geeraerts, 2009, p. 822) and taking a concept-led approach. At a basic level, we operationalize *concept* as a semantic category represented by a cluster of synonyms and hyponyms. In the current work, the categorisation of words into concepts, conceptual hierarchy, and concept labels derive from WordNet (Fellbaum, 1998, more discussion in Section 3).

Previous research in conceptual variation (e.g., Mehl, 2021; Fitzmaurice, 2022; Fitzmaurice and Mehl, 2022; Robinson and Weeds, 2022) has attested the value of using concepts to explore patterns of cultures, thoughts, and behaviours. For example, Fitzmaurice et al. (2017, p. 21) showcase the ways in which “key cultural concepts” provide insight into the diachronic trends in the shaping of thought, culture, and society by analysing the concept of valor. Robinson and Weeds (2022) explore gendered speech in the Old Bailey Corpus to discover the phenomenon of *socio-conceptual polysemy*, which indicates that different people may use the same concept with the same probability but develop different meaning components for that concept.

The key research question that drives the present endeavour asks which concepts are distinctive of the COVID-19 era in relation to the previous decade. We address this question by analysing longitudinal data from a corpus of day diaries collected by Mass Observation Archive and written on the 12^th^ of May on each of the years from 2010 to 2020 (Massobs, 2010). The diary writers respond by email or letter to the same instruction to account for everything they did on the 12^th^ of May of the given year. Because the data is consistent in terms of the context of language use and topic, the data yields itself well to the comparative analysis across time.

In order to find similarities and differences between two texts, corpus linguists typically employ the keywords approach (e.g., Baker, 2004; Love and Baker, 2015; Hansen, 2016). A *keyword* is a word which occurs in a text more often than we would expect to occur by chance alone. Keywords are calculated by statistical tests which compare the word frequencies in a text against their expected frequencies in a reference corpus. In the current research we propose taking the perspective of a *keyconcept* as a beneficial way of identifying trends in broader patterns of cognition. A *keyconcept* is similar to a *keyword* in terms of it extracting a topic that is typical of a given text against the idea in a reference text. A *keyconcept* differs from a *keyword* in that it captures that idea not through an individual word but as a concept, i.e., a group of semantically similar words. While we employ this approach and showcase its efficacy in this article, it is important to note that we also acknowledge that concepts are represented by more complex structures of language and cognition, but this broad view of concepts is beyond the remit of the current paper (see Murphy, 2004).

The focus on concepts in language enables us to explore broader patterns of thinking from a given text. We identify concepts which reflect lived-experiences that are particularly prominent in the COVID-19 era. We do so by conducting quantitative and qualitative analysis of a longitudinal corpus. Firstly, we conduct quantitative analysis in order to identify which concepts are most distinctive of the COVID-19 era. We then analyse the data qualitatively, in order to establish how these distinctive concepts are being used and how they relate to the ontology of COVID-19 in the United Kingdom.

In Section 2, we present details of the data used in the current study, alongside an overview of Britain on the 12^th^ of May 2020, the day in which the target dataset was collected. In Section 3 we provide an overview of the method, before introducing the case-study of the keyconcept regulation. In Section 4 we interpret the sentences containing the concept of regulation in a discourse analytic framework (cf. Van Leeuwen, 2008). The discourse analysis yields themes of agency, emotions, stance, hearer-new information, and temporal framing of the concept of regulation. In the final remarks, we comment on the key findings of the discourse analysis and usefulness of the keyconcept approach taken in the study.

## 2. Data

### 2.1. May diaries and the Mass Observation Archive

The Mass Observation Archive (MOA) “specialises in material about everyday life in Britain. It contains papers generated by the original Mass Observation social research organisation (1937 to early 1950s), and newer material collected continuously since 1981 (Mass Observation Project)” (http://www.massobs.org.uk/). Since 2010, the MOA has made an annual call for day diaries written on the 12^th^ of May by self-selected members of public. The diarists are instructed to record everything they did from the moment they woke up in the morning to the time when they went to sleep on the 12^th^ of May and add “any reflections on the day [12^th^ May] and how you [they] felt while keeping the diary”. Guidance for respondents is also provided relating to the submission of biographical information as well as confidentiality and anonymity. This means that there is minimal top-down interference in the content of these diary entries. Thus, the responses can be considered to be reflective of the lived-experiences and concerns of the diarists on the day of writing. While press releases relating to the 12^th^ of May diaries have differed since 2010, the core guidance for diarists has remained stable (http://www.massobs.org.uk/write-for-us/12th-may).

While diarists are free to handwrite their responses and to include additional materials such as photographs or drawings, the scope of the current analysis is limited to digitally-submitted written responses. As almost identical instructions have been provided to diarists each year for the 12^th^ May project, we assume that differences across each year's responses reveal information regarding the distinctive lived-experiences of each particular year. This homogenous structure of the data enables us to query distinctive concepts of the diaries from the 12^th^ of May 2020, during COVID-19 lockdown, against the baseline of the pre-COVID-19 diaries from 2010 to 2019.

Little research has made use of the 12^th^ of May diaries so far. An exception to this is Langhamer (2020) who investigates the May 2020 diaries and identifies a number of key themes such as a sense of living through history. To the best of our knowledge, no diachronic comparative analysis has previously been conducted on the 12^th^ of May diaries. We next describe the data and research methods, before contextualising Britain on the 12^th^ of May 2020 and then analysing the results of the keyconcept approach to the 12^th^ of May diaries.

### 2.2. 12^th^ of May diaries: Corpus characteristics

**Figure 1:**
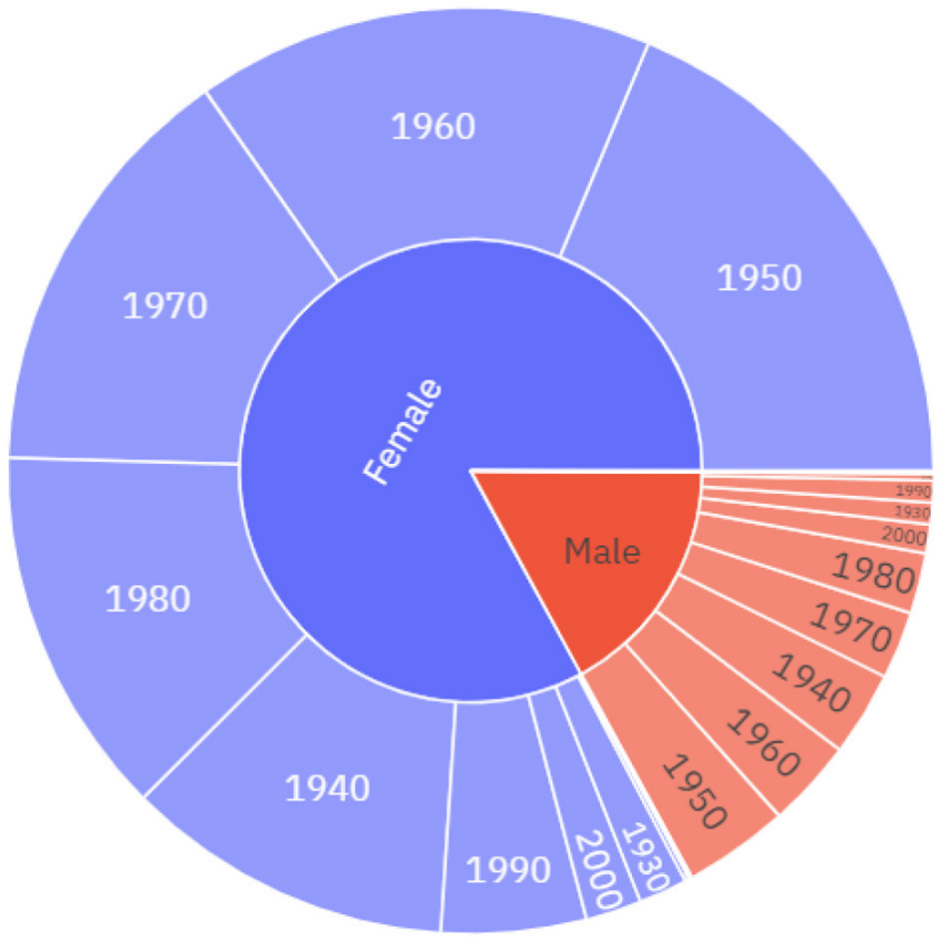
The socio-demographic distribution of the diarists, 2010–2019, by gender and decade of birth.

In this section we present the properties of the 12^th^ of May Diary Corpus used in the current research project including socio-demographic characteristics of diarists based on the information available regarding their age, gender, location, and occupation.^1^ The 2010–2019 diaries include 3,070 diary entries and 4,101,605 words, with an average length of 1,336 words per entry. Between 2010 and 2019 the average number of responses to a 12^th^ of May diary was 307, with the highest response rate being 582 in 2016 and the lowest being 142 in 2019. In the 2010–2019 diaries, excluding some cases, such as those where the gender was not provided, 82.2% of the diarists identified as female and 17.7% identified as male. The 1950s–1980s are the most common decades of birth for these diarists, with minor differences between males and females (see Figure 1).

**Figure 2:**
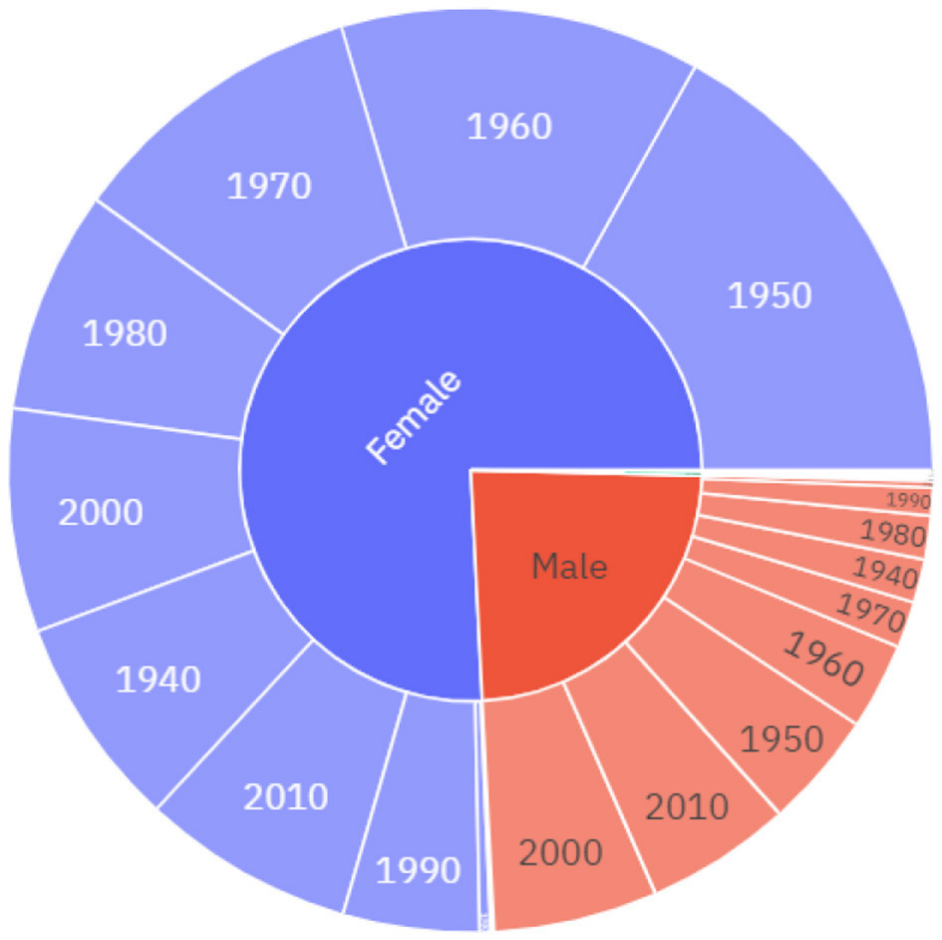
The socio-demographic distribution of the diarists, 2020, by gender and decade of birth.

The 2020 dataset includes 4,478 diary entries and 4,921,831 words, with an average length of 1,099 words per entry. A comparison of the quantities of data across the datasets reveals the unprecedented response rate to the May 2020 diaries. There were almost one and a half thousand more responses in 2020 alone than in the previous 10 years combined. In the May 2020 diaries, 75.9% of May 2020 diarists identify as female, and 23.7% of diarists identify as male. While there is no clear skew towards female respondents in May 2020 (Figure 2) this skew is smaller than in the 2010–2019 diaries (see Figure 1). The mean age of diarists in May 2020 is 45 which is slightly younger than the average ages from the previous decade of diaries, which range from 45 in 2013 to 55 in the 2019 diaries. The downward skew on diarists ages in May 2020, particularly among males (see Figure 2), can largely be attributed to the fact that a number of school classes that participated in the 12^th^ of May diaries was much higher in 2020 than in previous years. There is also a slightly different age-profile between male and female diarists, with males born in the decades 2000s and 2010s making up a much larger proportion of the male diarists in relation to the female diarists. While all major geographical areas in the UK are represented in the data, there is also a skew towards those diarists from London and the South-East, as well as from higher socioeconomic backgrounds.

The diaries represent the universe of thoughts, behaviours, and cognition of the writers contributing to the data (cf. Hubble, 2006; Savage, 2007). Admittedly, these contributors are disproportionately skewed towards middle-aged women from the South East of England. However, these demographic skews are not specific to the 2020 dataset analysed here, but are consistent across the entire MOA. Thus, the conclusions related to salient themes across the 2010–2019 and 2020 datasets can be considered representative to the same degree that any other analysis that uses the MOA can be. While the rich biographical information regarding the diarists means that the data yields itself to an analysis of differences in the diaries according to socio-demographic profile, such an analysis is beyond the scope of the current paper.

### 2.3. Context: Britain on the 12^th^ of May 2020

According to the Office for National Statistics (2021), the first wave of Coronavirus in the UK began in March 2020 and ended at the end of May 2020 (WordNet, 2010). Britain reported its first case of Sars 2 COVID-19 on the 29^th^ of January 2020. After a period of time when the government tried to control the spread of the virus with behavioural guidelines, such as recommendations to replace handshakes with elbow bumps and to wash hands for the duration of two iterations of the happy birthday song, the UK entered a lockdown on the March 26^th^, 2020. This meant that all those who could were ordered to work from home and leaving the home was permitted in only very specific circumstances such as for daily exercise or to buy essential goods such as food (House of Commons Library, 2021). In England, the relaxation to lockdown restrictions was announced by Boris Johnson on the May 10^th^, 2020 (it was announced in Parliament the following day), while in Scotland and Wales the lockdown dates and guidance differed slightly. The UK government permitted two people from different households to meet outdoors from the May 13^th^, 2020, the day after the 12^th^ of May diaries were written. The 12^th^ of May 2020 predates the availability of COVID-19 vaccines for the public, with the first coronavirus vaccine outside of clinical trials being administered in December of 2020.

## 3. Methods

In order to discover which concepts are distinctive of the COVID-19 era in relation to the previous decade we analyse data from day diaries written on the 12^th^ of May 2020 and compare with the same data produced in 2010–2019. We refer to these two corpora as May 2020 and May 2010–2019 corpora, respectively. The current analysis is based on a distant and close reading of the data. First, we identify concepts which are distinctive of the target dataset, which is May 2020, in relation to baseline data of the May 2010–2019 diaries. Once the keyconcepts are identified, we apply traditional corpus and discourse analytic techniques to the analysis of a sentence containing the lexeme representing the keyconcept and, occasionally, the immediate context of that sentence.

### 3.1. Computational approach

We have developed a pipelined approach to keyconcept extraction which follows the four steps, i.e.:

1. Corpora creation
2. Word sense disambiguation
3. Automatic concept annotation
4. Keyconcept extraction

In Step 1 we create the target corpus for May 2020 and a reference corpus for May 2010–2019. The diary responses submitted to MOA in digital formats were converted into .docx files. The files were cleaned, anonymised, and tagged for meta-data such as gender, age, region, and occupation. Ethical and legal approvals to work with the MOA data have been obtained by Authors. In Step 2, each word in the corpora is tagged for part-of-speech and sense using Supervised Word Sense Disambiguation (SupWSD) (see Papandrea et al., 2017). In Step 3, we use WordNet 3.0 (www.wordnet.princeton.edu) to position each sense in a hierarchy consisting of semantically more general and more specific senses. Thus, words which share meaning are grouped by means of conceptual-semantic and lexical relations, such as synonymy or hyponymy. The resulting network of semantically-related words creates a concept. In Step 4, we extract the keyconcepts that are distinctive of the target dataset.

**Figure 3:**
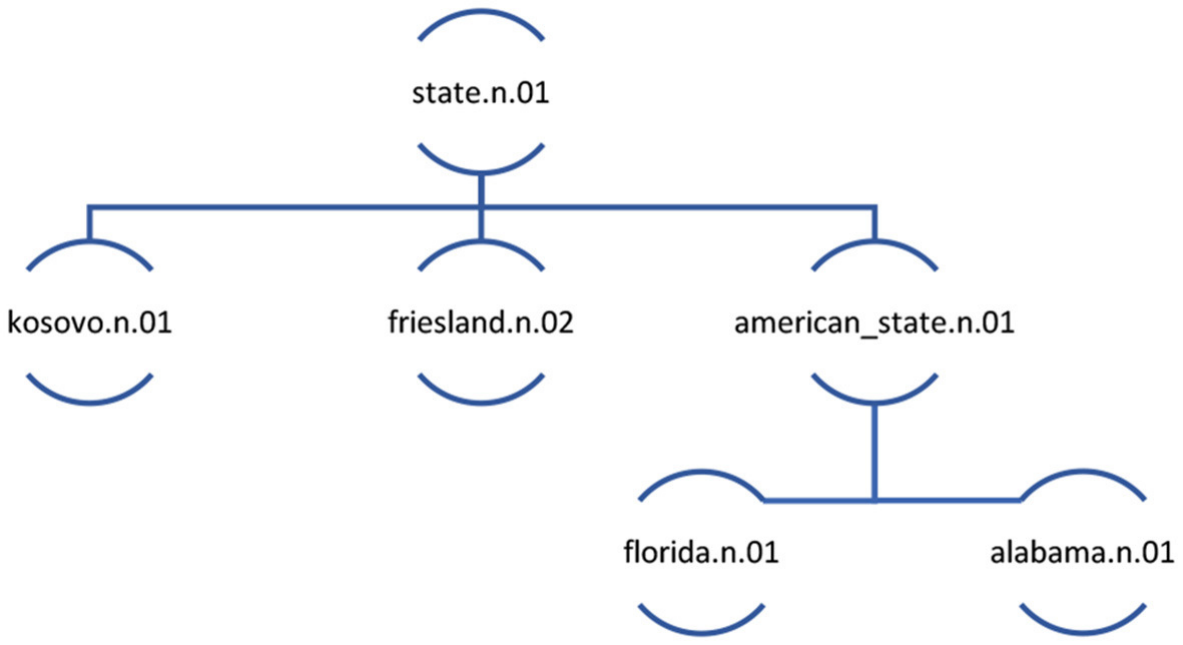
The abbreviated representation of the concept of state.n.01.

We acknowledge the principles on the basis of which WordNet reifies concepts (cf. Fellbaum, 2005; Jezek and Hanks, 2010). Certain nuances of language use are missed when lexis is aggregated into conceptual entities proposed by WordNet or other knowledge-based ontologies. In this paper we show the value of aggregation of words into concepts, such as the one that emerges from the point of view of computational handling of data. We also zoom in on nuances of language use by carrying out discourse analysis of a statistically meaningful dataset. Thus, we reconcile distant and close reading of the texts. In order to do so, first, it is necessary to clarify a key terminological distinction between *senses* and *concepts*. As a result of steps 2 and 3 each word gets assigned a particular sense, labelled by a word form which represents its meaning, a letter signifying part of speech category (e.g., n = noun, v = verb), and a number referring to distinct polysemous meanings of the word form. For example, the noun *state* “the territory occupied by one of the constituent administrative districts of a nation” is represented as state.n.01, while state.n.02 is defined as “the way something is with respect to its main attributes”. The sense state.n.01 consists not just of the noun *state*, but also its (near) synonym, the noun *province*. state.n.01 can also be seen as a concept, that is, an abstraction which includes state.n.01 as well as hyponyms of state.n.01, including american_state.n.01, kosovo.n.01, and friesland.n.02. A concept also includes senses further down in the semantic hierarchy. Thus, the hyponyms of the hyponyms of state.n.01^2^ are also included in the concept state.n.01 (Figure 3). The main benefit of focusing the analysis on the concept is due to its capacity to capture themes in texts represented by a whole group of semantically-related words.

WordNet structures concepts into a taxonomic hierarchy, which is defined by hyponymous or *is-a* relationships. All nouns begin with the “beginner synset” entity.n.01 at level 0 which has a range of hyponyms, which themselves have hyponyms and so on, recursively, until very specific results such as lefteye_flounder.n.01 appear at the 15^th^ level in the taxonomy. The example of the concept of state in Figure 3 illustrates the hierarchy and sample levels, where state is at level seven in WordNet's conceptual hierarchy.

In Step 4, we identify keyconcepts through the use of Pointwise Mutual Information (PMI). Specifically, we use PMI to determine which concepts are distinctive of the target corpus (May 2020 diaries) in relation to the reference corpus (May 2010–2019 diaries). PMI is a commonly used metric which measures strength of association (e.g., Hoang et al., 2009; Evert, 2008). While PMI is often used to determine the likelihood of two words occurring next to or within a specified window of each other (e.g., Lai, 2019; Hilpert and Flach, 2021), PMI can also be used to investigate how much more likely a word or a concept is to occur in one dataset in relative to another dataset. The relative frequency (tokens of the concept measured against total tokens in the dataset) of a given concept in the target corpus is measured against its relative frequency in the baseline. In the current study the PMI measures the strength of association between a concept and May 2020 diaries against the expected association of the that concept with May 2010–2019 diaries.

We use the following equation to determine PMI:


$$\begin{array}{l} \text{PMI}\left(A,B\right)=log\;\frac{P\left(A|B\right)}{Pref\left(A\right)} \end{array}$$

where A is a concept, B is a directive, P(A|B) is the probability of encountering concept A given a directive B, and Pref(A) is the probability of concept A in the reference corpus.

## 4. Results

### 4.1. Diarists on May 12^th^, 2020

The lived-experiences of this first UK lockdown are preserved in the form of the MOA's 12^th^ of May diaries from 2020. The diarists were asked to record everything they did from the moment they woke up in the morning to the time when they went to sleep on the 12^th^ of May. These diaries contain detailed descriptions of lockdown life on the day as well as narratives relating to differences between their routines pre/during lockdown. When compared to the diaries in the previous decade, we expect to discover concepts that are distinctive of 2020. We expect many of those concepts to correspond to the salient memories of pandemic life. We also expect to find concepts statistically distinctive of 2020 but less salient to the memory of pandemic life.

**Figure 4:**
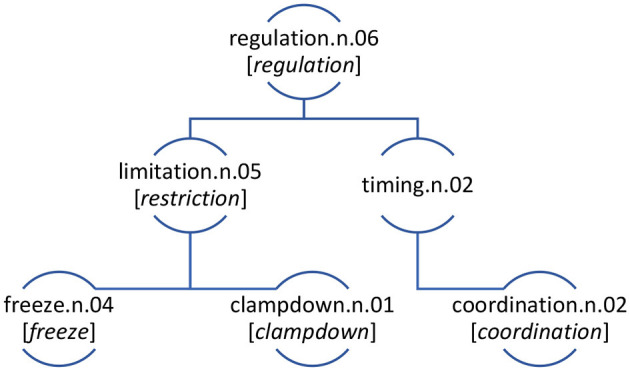
The concept of regulation in the 12^th^ of May 2020 Diaries with the lexemes used to express the concept at different levels in brackets.

The computational analysis of May 2020 allows us to query the dataset and identify areas of distinctiveness that we operationalize through the idea of the keyconcept. The concepts that are most distinctive of May 2020, compared with May 2010–2019 include the first ranked most distinctive concept of lockdown.n.01 (*n* = 11,511; PMI = 13.22), the forth ranked concept soar.n.01 (*n* = 1,875; PMI = 10.60), the fifth ranked concept pandemic.n.01 (*n* = 2,486; PMI = 9.43), the seventh ranked concept furlough.n.01 (*n* = 570; PMI = 8.89), and the ninth ranked concept distance.v.01 (*n* = 517; PMI = 8.75). Many of the most distinctive concepts are largely intuitive considering the memory of the pandemic life. Most concepts have a high-degree of name agreement (see Snodgrass and Vanderwart, 1980), that is, there is one word or a small set of words used to lexicalize that concept. For example, the 570 times the concept furlough.n.01 was used, it was realised exclusively by the lexical item *furlough*. In such cases, the results from the keyconcept analysis do not differ greatly from more traditional keyword analysis using corpus methods (e.g., Love and Baker, 2015; Hansen, 2016). However, other highly distinctive concepts of May 2020 diaries are represented by a range of lexemes and, therefore, showcase the value of focusing the current analysis at the level of the concept. For example, the concept regulation.n.06 (henceforth regulation), which WordNet defines as “the act of controlling or directing according to rule”, is comprised of a range of different lexical items. Specifically, regulation, which has a PMI of 6.85 was used 49 times in the May 2020 diaries^3^ of which there were 29 uses of *restriction*, 11 uses of *freeze*, seven uses of *coordination*, one use of *clampdown* and one of *regulation*.^4^ The keyconcept of regulation presented in Figure 4 is the focus of remaining part of the current paper. The concept of regulation (regulation.n.06) is 54^th^ top nominal keyconcept across all data as ranked by PMI. Although it is not the top ranked most distinctive concept in the data its structure and usage provide a useful case study to illustrate the method proposed in the current paper.

The WordNet at levels in the concept hierarchy are as follows, i.e., regulation.n.06 is at level 8, with limitation.n.05 and timing.n.02 being at level 9, and clampdown.n.01, freeze.n.04, and coordination.n.02 being at level 10. While it is clear how most of the lexical items within the concept of regulation map onto the senses in Figure 4, it is worth noting that *restriction* is tagged as the sense limitation.n.05.

The social distribution of regulation in the dataset broadly reflects the socio-demographic profile of the May 2020 diarists (Figure 2). For example, the gender distribution, excluding unknowns, was approximately equal to that of the dataset as a whole, with 22.22% of those who used regulation identifying as male and 74.07% as female. However, the users of the 49 instances of the concept of regulation have an average age of 59, which is older than the May 2020 diarist average age of 45.

While the computational methods serve to identify keyconcepts, close reading of the data enables a more nuanced understanding of the identified concepts. In order to explore the usage of the concept of regulation, we move to the corpus-assisted discourse analysis of data.^5^ We extract and discuss themes common across all the terms of regulation rather than presenting the semantics of each term separately. The analysis focuses on the sentences that contain the terms of the concept of regulation, sometimes using surrounding sentences to provide necessary context.

### 4.2. Discourse of the concept of regulation

Following the computational analysis, in this section we take a discursive perspective based on Van Leeuwen's (2008) analytical framework to investigate the immediate context in which the keyconcept regulation is used in the 12^th^ of May 2020 diaries. Van Leeuwen's (2008) approach considers discourse as a representation of reality. Thus, different narratives may capture the same facets of reality in different linguistic ways and to different purposes. Such analysis makes it possible to discover implicit or explicit ideas and stances associated with regulation.

In what follows we highlight features of the language used in the contexts of words of regulation and illustrate them with excerpts from the diaries. The entries which we discuss reflect the writers' stances associated with the pandemic and the “unprecedented” (as the buzz qualifier used in many media reports) state of affairs. The diarists note down the major social event responsibly and diligently. We organise the current section along the key discursive characteristics present in the context of the concept regulation which include themes of agency, emotive language, the particular use of pronouns, use of existential constructions, temporal deixis and narrativization of experience. Together these themes provide the sense of how the diarists felt when dealing with COVID-19 regulations.

#### 4.2.1. Agency of regulation

The construct of agency is ubiquitous as is controversial and difficult to define. In the “skeletal” definition by Ahearn (2001, p. 112), “[a]gency refers to the socioculturally mediated capacity to act”. While such a basic definition leaves many questions open, we understand *agency* as intrinsically historical and situated, and the reference to the ability to act as the capacity to choose social practises of a particular kind and/or discern the type of discourses one wants to use (Bacchi, 2005). In the diaries, the first notable strategy that reduces the agency of the writers is their tendency to transform social actions into objects according to the processes that Van Leeuwen (2008, p. 63–66) terms *objectivation* and *descriptivation*. Actions can be objectified, if represented statically as nouns and they can be descriptivised, if represented as permanent qualities. Together with nominalization, i.e., the reduction of a verb phrase to an abstract noun, these strategies are ways to hide or disguise the underlying processes indicating who does what and reduce the subject's authority/responsibility (Hart and Fuoli, 2020).

A prominent instance of objectivation in the context of the concept of regulation is the metaphor of *freeze* in the occupational field. This metaphor summarises the experience of lives being suspended and very differently regulated during the pandemic, as in Examples 1–4. Writers comment on remuneration at work being at a stall, changes to responsibilities and work tasks. These excerpts suggest that freezes in the occupational domain are an integral composite of the broader salient theme of regulation of the first lockdown.

1) “We're working at half capacity due to a recruitment freeze so it is just me and my news editor producing three to four storeys a day about the insurance industry”.
2) “Work is also difficult because of the financial crisis that universities find themselves in—restructuring and redundancies loom, supporting tutors (especially PhD students) are on a hiring freeze, along with research time, sabbaticals, promotions”.
3) “I was recently promoted at my current job (no pay rise though as there is a pay freeze)”.
4) “I wouldn't usually oversee this function but with a recruitment freeze across the university, I have volunteered to line manage this team as the team head's recruitment has been paused”.

In Example 5, the verbal nominalization of *easing of restrictions* hides the Government decision to relax COVID-19 rules. This grammatical construction contains no explicit reference to any actor or agent (Van Leeuwen, 2008, p. 30). It represents the restrictions, even if they are relaxed, as an overhauling entity that still regulates people's lives. Similarly, in Example 6 the descriptivised action of *demands of considering three or four different ways of delivering learning* reduced to a nominal phrase is something that hovers over the locked-down citizens depriving them of any agency. Other expressions in Example 6 confirm and support the sense of general incapacity to reappropriate control that the diaries convey. Examples range from the use of prepositional construction suggesting imposition as in *demands are (…) upon us*, to proper nominalizations such as *hiring freeze*, and the use of noun phrases as in *complete uncertainty*, which all remove reference to a particular agent or actor. Also, the use of the passive voice in *the teaching would be delivered* (Example 6) and in *this role has now been suspended* (Example 7) suggests the reduction of choices and de-agentivation of people (Van Leeuwen, 2008, p. 23–74) under COVID-19. The entity who carried out the action is not specified.

5) “Current easing of restrictions which state that members of different households can meet up one on one means that only one of us could meet one of our little grandchildren on their own if we could get to London without using public transport and not stay overnight—a non-starter”.
6) “The end of March was filled with panic for students and staff, this eased a bit in April but then the demands of considering three or four different ways of delivering learning in September is upon us with a hiring freeze and complete uncertainty regarding whether students would return to study next year if teaching would be delivered only remotely due to the need to social distance for safety”.
7) “This role has now been suspended due to the Coronavirus pandemic, associated health risks and travel/quarantine restrictions”.

The deagentivation of COVID-19 social actors as in the use of nouns *availability* and *delivery slots* is exemplified in Example 8. However, in this example, the nominalization of *coordination* is presented as regulation among neighbours over themselves or each other, which differs from the regulations presented in the previous examples whereby an unnamed authority is responsible for the regulation. Thus, the phrase of *coordination between neighbours* could be an example of grassroots agency.^6^

8) “It has sometimes been difficult obtaining fresh fruit and vegetables in recent weeks but a combination of better availability at the local Tesco Express, greater choice of grocery delivery slots and friendly cooperation and coordination between neighbours over shopping has improved the situation greatly”.

A greater agency behind regulations is visible in Examples 9–11. It is *Boris Johnson, Wales*, and *the UK* that are the subjects in active constructions, and they have agency over the restrictions. Other examples of agents in the example of regulation include mainly institutional agents such as *ministers, collective political, large grocery suppliers, universities, The National Trust*.

9) “Boris Johnson has announced slight lessening of the restrictions”
10) “Wales have extended the restrictions”
11) “The UK has imposed the restrictions”

Within a context in which people depend upon the development of the pandemic and the decisions made by others in the name of safety, individuals' perceived freedom seems curtailed, especially in the sphere of employment. Active and identified agency used in the context of regulation is mainly assigned to institutional actors.

#### 4.2.2. Emotions of regulation

Another aspect of the language surrounding the concept of regulation in the diaries is the reference to affect (Martin and White, 2003). Uses of regulation contain socially-constructed feelings that convey the diarists' limitation or loss of agency. In the phrase *I am grateful* In Example 12, the writer as “emoter” thanks someone who is the agent who carried out an action while in Example 13 the emoter is scared of the decision to lift the restrictions.

12) “I am grateful for the coordination between the government and large grocery suppliers that has enabled this to be the case”.
13) “I fear that [lockdown restrictions] may have gone too early and England would have done better to keep the restrictions unchanged for another 3 weeks as have the other home nations”.

In Examples 14 and 15, the direct reference to emotions points to the situation haphazardness and/or the dissatisfaction associated with the writers' abandonment to the uncontrollable forces dictating their lives as in the phrases *I feel very lucky/I felt almost guilty/I feel this is dangerous*. In all these cases, the feelings are “construed as directed at or reacting to” (Martin and White, 2003, p. 47) the COVID-19 regulations, and the limitation of choices that the unprecedented situation brings.

14) “This last weekend, prime minister Boris Johnson has announced slight lessening of restrictions on movement, but I feel this is dangerous, muddled and confusing thinking”.
15) “The loosening of restrictions has created so much confusion and a breaking of the 4 nations approach and it feels like people are once again being thrown to the wolves”.

Even in the very different situation when people do not critique regulations, but in fact welcome them, diarists' language encodes the uneasiness associated with safety decisions being made by others. In Example 16 one diarist discusses their fears at the easing of restrictions and states that they are going to self-impose an extension of the lockdown restrictions. In Example 17 the diarist reflects that their access to space relieved them of the pandemic limitation. In both cases, the writers decide not to bow to the government decisions that “allows” them to put an end to the “stay at home” regulation. On the contrary, fearing the virus, they prefer to continue in lockdown.

16) “tomorrow […] the government are allowing some people to go back to work and encouraging longer outings for exercise. It doesn't feel safe yet as I'm 69 (70 later this year) I'm going to carry on with the first lock down restrictions until I feel comfortable with going out and going further afield”.
17) “I have been spared many of the difficulties of the restrictions. I even have a garden to enjoy. I do feel very fortunate”'.

Incidentally, the diaries express an expected socioeconomic disparity through the writers' different access to space (see also Howlett and Turner, 2022) as a consequence of the pandemic regulations. Diarists reflect on their relative privilege of having access to a range of spaces, particularly outside spaces and spaces which are conducive to working from home as in Example 17. There are also comments judging the restrictions as not very strict as in Example 18.

18) “The restrictions of the pandemic have not seemed too harsh”.

Feelings can also be expressed through adverbs that can function as “interpersonal theme” (Halliday, 1994). They encode the writers' dependency on fortuitous events as in *[t]hankfully restrictions have slowly eased up* in Example 19. The diarist's gratitude reflects their feeling of dependency on somebody or, rather, something that accidentally produces a positive result they are incapable of achieving. Both the predicative adjective *fortunate* in Example 18 and the adverb *thankfully* in Example 19 encode the haphazardness of the situation in which people have a limited agency.

19) “From mid March, countries across the world including where I live [the United Kingdom] went into strict lockdown, thankfully restrictions have slowly eased up”.

The analysis of the immediate contexts surrounding concept of regulation indicate emotional reactions to regulations, whether imposed externally or self-imposed. These reactions mainly encoder fear, but also gratitude for being spared inconveniences of imposed regulations.

#### 4.2.3. Stance and novelty of regulation

When talking about regulation, the diarists often take a collective stance through the use of the plural pronoun *we*. The analysis of the pronoun *we* requires going beyond “a grammatical point of view to engage with the semantic and pragmatic levels” (Goddard, 1995, p. 99). Therefore, it is through a close consideration of the context that one can establish the extent to which the plural pronoun is the equivalent of to the pronoun *I* (Ige, 2010) or truly reflects a community as in the case of Parliamentary communities, see (Íñigo-Mora, 2004) or in political movements (Lee et al., 2020). In the diaries, the pronoun *we* underlines the sense of a community of individuals sharing the experience of regulations in the same time and space. The extracts in Examples 20–22 show the switch from a typical-diary style use of *I* to the collective *we*. The diarists' use the pronoun *we* encodes an individual experience which is shared with the other people. Diarists resort to taking on the task of reporting for the nation/community.

20) “Taxes, wage freezes and pension adjustments will most likely be the route back to financial stability—we accepted that repayment must be made but are worried about the force of the hit on the population, young and old”.
21) “Difficult to see how things will look once we all emerge from restrictions”.
22) “Wales have extended the lockdown, but we know that day trippers will start to arrive in the country shortly, and the new restrictions are impossible to enforce”.

Reporting on new information while talking about the concept of regulation is also evident in the analysis of the existential *there* construction. The discourse-pragmatic function of existential *there* sentences is “to introduce the NP [noun phrase] referent into the discourse world of the interlocutors by asserting its presence in a given location” (Lambrecht, 1994, p. 179). That referent must be hearer-new, and this requirement has been expressed by an explicit “Novelty Condition” on the entity introduced by the existential construction (cf. McNally, 1992; cf. also Abbott, 1993, 1997; Ward and Birner, 1995; Cruschina et al., 2012). In the context of the concept of regulation there are numerous examples of new information introduced by the existential *there* constructions. Some include such phrases as *fear and anxiety, pay freeze, queries about lockdown measures, parallels to be drawn in terms of restrictions on personal freedom, no evidence of any proper coordination* as in Examples 23–27.

23) “there's clearly fear and anxiety about the (slight) easing of restrictions”
24) “there are parallels to be drawn in terms of restrictions on personal freedom”
25) “no pay rise though as there is a pay freeze”
26) “There are numerous queries about lockdown measures, especially in the wake of Boris Johnson's announcement at the weekend easing (slightly) restrictions in England”.
27) “There has been no evidence of any proper coordination of action for this global crisis”.

The use of collective *we* and existential *there* constructions in the context of the concept of regulation reinforces the collective experience of the pandemic, the novelty of the situation, and the need of diarists to capture the observable reality of regulations.

#### 4.2.4. Narrativising the regulation

At times diarists “narrativise” (Gee, 1985) their experience of regulation whether by presenting their storey in isolation or in relation to experiences of other people. In most cases these texts present their own experience, whether partial or full, as organised, logical and sharable. Rather than being approached as perfectly organised structures with a beginning, a climatic middle and an end (Labov, 2010), these small storeys are appreciated in their being fragmented, essential, even incomplete narratives (Bamberg, 2006; Georgakopoulou, 2006) that share a number of features. Besides the canonical use of past tense to report on actions and event that took place and that the diarists witnessed or experienced these small narratives show other chronological realisations.

In some extracts of regulation the chronological dimension is realised through the construction of hypothetical narratives, e.g., *I'd go and stay with my family* in Example 28 or production of accounts of attempted actions, *my husband has arranged to play golf* in Example 29. Alternatively, the narrative experience is reduced to a list of habitual essential events, such as *dressed (…) washed the pots and then there was a delivery* in Example 30. In the last clause of Example 30 is worth noting the switch from an implied “I” to a less personalised form with *there* (cf. Section 4.3.3).

28) “I also said that when restrictions were relaxed, I'd go and stay with my family as soon as possible”.
29) “My husband has arranged to play golf on Thursday with 1 other person and masses of restrictions”.
30) “Up, dressed and breakfast of toast and more tea, watched the news (still mostly about the Corona virus and changes to restrictions which kick in tomorrow) on BBC Breakfast, washed the breakfast pots and then there was a delivery from the post office”.

Because of COVID-19 regulations, time becomes difficult to manage. Individuals declare their inability to act as in phrases *I will not be able to be on the rota* (Example 31) or *It has sometimes been difficult obtaining fresh fruit and veggies* (Example 32).

31) “I will not be able to be on the rota to babysit any more due to restrictions on meeting”.
32) “It has sometimes been difficult obtaining fresh fruit and vegetables in recent weeks but a combination of better availability at the local Tesco Express, greater choice of grocery delivery slots and friendly cooperation and coordination between neighbours over shopping has improved the situation greatly”.

In other storeys, regulations become the new measure of time. A striking number of examples of temporal deixis accompanies the concept of regulation (see Example 33). While references to immediate time (hours and days) in diary writing are expected, references to larger time frames are striking as the diarists were asked to record what they did on 1 day, i.e., the 12^th^ of May. Diarists frame the concept of regulation in relation to weeks *(next few weeks, recent weeks, last two, three, six, seven weeks)*, months *(two months, March, 23 March, mid-March, April, June, July, September)*, year *(next year)*, the future *(future travel, immediate future)*.

33) “I also said that when restrictions were relaxed, I'd go and stay with my family as soon as possible—at one point we thought maybe in June, now it's looking like July at the earliest—but now I'd be concerned about staying with them while my brother is going out to work”.

The events that co-occur with the concept of regulation and a wider time frame include the length of lockdown, as well as seeing family, and staying with family. Diarists also talk about the new reality of working from home delivering learning, hiring freeze. They occasionally mention travel for leisure.

As for the use of *yesterday* and *tomorrow*, diarists take the reader through the historical events of that time by noting Johnson's announcements of lockdown restrictions “yesterday” (11^th^ May) as in Example 34 or changes in in lockdown restrictions from “tomorrow” (that is, 13^th^ May) in Examples 35–38.

34) “We usually chat about our plans for the day but yesterday's announcement by the PM about lockdown restrictions being relaxed in England means that both my husband and I are distracted and looking at our phones whilst necking coffee in the kitchen”.
35) “from *tomorrow* we'll no longer be able to enjoy walking on the local golf course as the golfers will be back with the ease up in restriction”
36) “there's clearly fear and anxiety about the (slight) easing of restrictions due to start *tomorrow*”
37) “restrictions are being eased from *tomorrow*”
38) “changes to restrictions which kick in *tomorrow*”

The use of temporal references in the context of the regulation and diary writing reflects how people organised rationally their experience of regulations to share with others. The analysis also shows that the concept of regulation becomes a frame for experiencing and talking about time.

## 5. Conclusion

In this paper, we discuss the 12^th^ of May diaries from 2010 to 2019 with the 12^th^ of May diaries from 2020, reflecting the differences between life before COVID-19 and life in the midst of the first wave of COVID-19 in the UK. We use computational methods to identify keyconcepts and the particular responses in which they occur before investigating the usage of these concepts. We focus on one of the most distinctive concepts in the 12^th^ of May 2020 diaries, namely, regulation, which was realised in texts by five lexical items, i.e. restriction, *regulation, clampdown, coordination*, and *freeze*. Following the computational analysis of this keyconcept, we engage in the contextualisation of regulation by offering a discourse analytical reading of a number of excerpts from the diaries and highlighting their linguistic features. The analysis shows that the keyconcept regulation is accompanied by the sense of limited individual agency and a dependence on abstract and uncontrollable factors or institutional actors. This is accompanied by tendency to refer to a language indicating feelings of fear and gratitude. These emotions are not solely triggered by the pandemic, but also by the novelty of the situation. The diarists record as much as they can perceive, conceptualise, and make sense of the lockdown. A lot of this reality is reported with gaps as to the agents and actors of regulation which is supported by impersonal constructions, fragmented narratives, or hearer-new information framing. Diarists make effort to report on the collective experience, for example, through the use of the pronoun *we* and make sense of the experience by a frequent reference to a temporal frame. At times, the responsibility of reporting on history takes precedent over reporting on their day. The diary task asked them to record everything they did from when they woke up in the morning to when they went to sleep at night on 12^th^ May, instead diarists often do not follow the brief, and use the diary writing as a tool for capturing the historical moment. In this context, diarists can be thought of as reporters or “citizen journalists” (Purcell, 2022) who provide a window on their world, the world of a contemporary society in real time. Their accounts of the concept of regulation demonstrate the sense of living through history consistent with Langhamer (2020) reading of the 12^th^ of May 2020 diary data.

The current study showcases the value of using keyconcepts to identify trends which represent salient lived-experiences. The traditional keyword approach does not flag up any of the lexical items analysed here as salient because individually they are not distinctive enough to meet required statistical thresholds (e.g., a “keyness” score). However, by broadening the focus from a word to a concept, we are able to demonstrate the salience of the entire semantically-related group of words that comprises the keyconcept of regulation. The keyconcept approach allows us to extract the concepts characteristic of the discourses as opposed to the words that are individually used to express those concepts. Additionally, through using conceptual hierarchies, we tap into the ontology of knowledge encapsulated, and reified by WordNet (cf. Fellbaum, 2005; Jezek and Hanks, 2010). Also, by engaging with discourse analysis, we demonstrate an appreciation and acknowledgment of textual nuances and, in doing so, reconcile the distant and close readings of texts. By considering the discursive contexts of use in which the concept of regulation appears in the May 2020 diaries, we show that a researcher remains critical in deciphering nuances from large datasets. Ultimately, the approach presented in the current paper successfully enables us to capture and explore diarists' thoughts and behaviours.

In addition to showcasing the value of concept analysis, we highlight the value of the MOA for social research. While the cross-disciplinary potentials of the MOA have been demonstrated by the diverse ways in which its data has been approached, we provide the first use of computational linguistic methods on the data. These methods and tools, such as PMI, showcase the value of the digital humanities in the context of identifying variation and change in attitudes and behaviours of the public.

## Data availability statement

The data used in the current research paper is available through the Mass Observation Archive http://www.massobs.org.uk/. Details of the data source and availability are also included in the article though data citations outlined in Section 2. Further inquiries can be directed to the corresponding author.

## Author contributions

All authors listed have made a substantial, direct, and intellectual contribution to the work and approved it for publication.

## Footnotes

^1^Note that this biographical information is shared at the respondents' discretion and, as a result, some elect to withhold part or all of it.

^2^Such as alabama.n.01 and florida.n.01 which are hyponyms of american_state.n.01.

^3^The concept regulation is distinctive of May 2020 diaries, not unique to them. This means that there are a small number of uses of this concept that appear, seemingly irrespective of COVID-19, such as “[c]oordination whilst looking in a mirror is very challenging”. There are also examples of regulation.n.06 in the baseline corpus, such as “[a]t the end of last year though, we had a 40% reduction in staff (following the coalition government's marketing freeze and cuts)”.

^4^49 instances of regulation.n.06 do not encompass all examples of the given lemmas that comprise this concept regulation. Many of these lexical items are polysemous and so are also tagged as constituents of other concepts. For example, other uses of *regulation* were tagged as regulation.n.01, “an authoritative rule”, and regulation.n.03 “the state of being controlled or governed”.

^5^While the concept analysis demonstrates that regulation was used quantitatively differently between the May 2020 and May 2010–2019 datasets, we do not engage in a comparative analysis of the discourses in which regulation occurs between these two datasets. We focus specifically on the discourses of regulation in May 2020, without a parallel analysis of the 2010–2019 data, which is beyond the scope of this paper.

^6^We thank Reviewer 2 for this suggestion.
